# Comparison of Apoptosis Detection Markers Combined with Macrophage Immunostaining to Study Phagocytosis of Apoptotic Cells in Situ

**Published:** 2007-02-07

**Authors:** Dorien M. Schrijvers, Guido R.Y. De Meyer, Mark M. Kockx, Arnold G. Herman, Wim Martinet

**Affiliations:** 1 Division of Pharmacology, University of Antwerp, Wilrijk, Belgium; 2 Department of Pathology, AZ Middelheim, Antwerp, Belgium

**Keywords:** apoptosis, phagocytosis, in situ detection, TUNEL, macrophages

## Abstract

Efficient phagocytosis of cells undergoing apoptosis by macrophages is important to prevent immunological responses and development of chronic inflammatory disorders such as systemic lupus erythematosus, cystic fibrosis and atherosclerosis. To study phagocytosis of apoptotic cells (AC) by macrophages in tissue, we validated different apoptosis markers (DNA fragmentation, caspase-3 activation and cleavage of its substrate poly(ADP-ribose)polymerase-1) in combination with macrophage immunostaining. Human tonsils were used as a model because they show a high apoptosis frequency under physiological conditions as well as efficient phagocytosis of AC by macrophages. On the other hand, advanced human atherosclerotic plaques were examined since plaques show severely impaired phagocytosis of AC. Our results demonstrate that the presence of non-phagocytized terminal deoxynucleotidyl transferase end labelling (TUNEL)-positive AC represents a suitable marker of poor phagocytosis by macrophages in situ. Other markers for apoptosis, such as cleavage of caspase-3 or PARP-1, should not be used to assess phagocytosis efficiency, because activation of the caspase cascade and cleavage of their substrates can occur in AC when they have not yet been phagocytized by macrophages.

## Introduction

Apoptosis or type I programmed cell death is an essential process during morphogenesis, tissue homeostasis and immune regulation and is involved in the elimination of infected, damaged or unwanted cells (Wyllie, 1992). The organelles and plasma membrane of apoptotic cells (AC) typically remain intact, avoiding proinflammatory spilling of the cellular content into the surrounding tissue. In this light, the apoptotic process has long been considered immunologically silent. However, recent evidence suggests that AC may also contribute to inflammatory processes via the release of proinflammatory cytokines (Hogquist et al. 1991) and/or chemotactic factors (Lauber et al. 2003). Moreover, inefficient removal of AC may lead to induction of postapoptotic necrosis and inflammatory responses. To prevent inflammation, cells signal their apoptotic state at an early stage to their environment, where they are recognized and engulfed by phagocytes (Fadok et al. 2001). Surface exposure of phosphatidylserine (PS) is one of these “eat-me” signals. To facilitate engulfment, apoptotic cells reduce their volume, contract their reorganized cytoskeleton and disintegrate into many small apoptotic bodies. Overall shrinkage is usually accompanied by nuclear condensation and internucleosomal DNA-fragmentation. Activation of the caspase family of cysteine proteases, in particular caspase-3, seems to be important in the latter process, as different caspase-3^−/−^ cell types, although still susceptible to certain apoptotic stimuli, show a complete absence of DNA fragmentation (Woo et al. 1998). Moreover, specific subsets of proteins are cleaved by caspase-3 and other executioner caspases to produce the apoptotic phenotype. Among the proteins cleaved during apoptosis by caspase-3, poly(ADP-ribose) polymerase-1 (PARP-1) is one of the main cleavage targets.

At present, cleavage of caspase-3 and PARP-1 as well as fragmentation of DNA are well known biomarkers for apoptosis in tissue. They can be detected either via immunohistochemical techniques (cleaved caspase-3 and PARP-1) or via terminal deoxynucleotidyl transferase end labelling (TUNEL) of apoptotic cells (DNA fragmentation). Because rapid recognition and clearance of AC by phagocytes is essential for normal development and prevention of inflammation and disease, phagocytosis of AC by macrophages has been extensively studied in chronic inflammatory disorders to fully understand their pathophysiological role. Accordingly, poor phagocytosis of AC has been identified in patients with systemic lupus erythematosis (SLE) (Ren et al. 2003), chronic obstructive pulmonary disease (COPD) (Hodge et al. 2003), cystic fibrosis (Vandivier et al. 2002) and atherosclerosis (Schrjvers et al. 2005). However, to interpret clearance of apoptotic cells by phagocytes correctly, appropriate detection markers are needed (Nakamura et al. 1996). Apoptosis in germinal centers of tonsils, as well as in lymph nodes, is a key regulatory event to eliminate non- and autoreactive lymphocytes. Nearly all AC in germinal centers of human tonsils are engulfed by macrophages, pointing toward a highly efficient recognition and elimination process of AC (Baumann et al. 2002). Phagocytosis of AC by macrophages in atherosclerotic plaques is severely impaired due to several factors, including cytoplasmic saturation, oxidative stress and competitive inhibition for common epitopes (Schrijvers et al. 2005). In the present study, we compared different in situ markers for apoptosis (caspase-3 activation, PARP-1 cleavage and DNA fragmentation [TUNEL]) to assess the efficiency of phagocytosis of AC by macrophages in non-inflamed human tonsils and advanced human atherosclerotic plaques.

## Methods

### Human specimens

Human non-inflamed hyperplastic tonsils (n = 8) were obtained from patients (mean age = 28 ± 9 years, 50% men) undergoing tonsillectomies. Human carotid endarterectomy specimens (n = 10) were obtained from patients (71 ± 3 years, 70% men) with a carotid stenosis of >70%. Specimens were fixed in 4% formalin within 2 min after surgical removal and paraffin embedded.

### Cell culture

In vitro experiments were performed using the murine macrophage cell line J774A.1 and the human monocyte cell line U937 (American Type Culture Collection). Cells were grown in RPMI 1640 medium supplemented with 10% fetal bovine serum, 100 U/ml penicillin, 100 μg/ml streptomycin, 20 U/ml polymyxin B and 50 μg/ml gentamycin. Cell culture media and supplements were from Invitrogen, fetal bovine serum was purchased from Sigma. To evaluate phagocytosis of apoptotic cells in vitro, J774A.1 macrophages and U937 cells were co-cultured in RPMI containing 50 μM etoposide. Previously, we demonstrated that etoposide (Sigma) induces apoptosis in U937 cells (77 ± 1% annexin V positive AC and complete cleavage of caspase-3 after 4 hours incubation without induction of necrosis (incorporation of propidium iodide)), but not in J774A.1 macrophages (Schrijvers et al. 2004). To prepare artificial cell blocks, cells were collected by scraping and centrifuged (900 × g, 7 min). Three drops of fibrinogen-rich plasma were added to the cell pellet. Subsequently, the pellet was detached from the tube using a wooden stick and three drops of thrombin (100 U/ml) were added. After shaking, cells were fixed in 6% formol for 1 h. The cell pellet was dehydrated using isopropylalcohol and toluol and paraffin embedded.

### Flow cytometry

Control and apoptotic U937 cells were stained with 10 μM of the cell-permeant DNA staining compound DRAQ5 (Biostatus Ltd) according to manufacturer’s instructions, and subsequently analysed by flow cytometry. Cell samples (20,000 cells/sample) were analyzed on a FACSort cytometer (BD Biosciences). Forward and side scatter gates were set to distinguish between viable and apoptotic cells. DRAQ5-emitted red fluorescence was monitored in the FL 3 channel after excitation of the probe at 488 nm. Data were analyzed using Cell Quest Pro software (BD Biosciences).

### Immunohistochemistry and terminal deoxynucleotidyl transferase end labelling (TUNEL)

Phagocytosis of AC by macrophages was examined by an indirect peroxidase antibody conjugate method using an anti-CD68 monoclonal antibody (clone PG-M1, DAKO) for macrophages, combined with an anti-cleaved caspase-3 polyclonal antibody (clone 67341A, Pharmingen) or an anti-cleaved PARP-1 p85 polyclonal antibody (Promega) to detect AC. The monoclonal antibody was detected using a goat-anti-mouse peroxidase secondary antibody (Jackson) for 45 minutes and visualized using Fast Blue as a chromogen. The polyclonal antibodies were detected by a PAP complex. For demonstration of the complex, 3-amino-9-ethyl carbazole (AEC) was used as a chromogen. Other primary antibodies used in this study were: anti-α-SMC actin (clone 1A4, Sigma), anti-vimentin (clone V9, DAKO) and anti-von Willibrand factor (The Binding Site Lt).

For the detection of oligonucleosomal DNA cleavage via TUNEL, tissue sections were pretreated with proteinase K for 10 minutes at 37°C and then rinsed with PBS. Subsequently, sections were incubated for 15 minutes at 37°C in a mixture containing 25 mM Tris-HCl, 0.25 mg/ml BSA, 200 mM potassium cacodylate, 5 mM CoCl^2^, 270 U/ml TdT (Roche), 10 μM dATP (Sigma) and 2.5 μM fluorescein-12-dUTP (Roche). Incorporated fluorescein-dUTP was demonstrated with a sheep anti-fluorescein peroxidase-conjugated antiserum (Boehringer Mannheim) at a dilution of 1/300 for 45 minutes and visualized by AEC. All TUNEL-positive AC in whole mount sections of plaques (n = 10) were counted and included in the results. Because of the high number of cleaved PARP-1 and cleaved caspase-3 positive AC in plaques, labeled cells in five random regions of interest around the necrotic core (120 × 100 μm each) were counted. In tonsils (n = 8), five randomly chosen germinal centers were evaluated for phagocytosis of AC. AC were considered phagocytized when they were surrounded by macrophage cytoplasm. Since binding of AC to macrophages not automatically results in uptake, bound cells were always considered not ingested.

To colocalize TUNEL with cleaved caspase-3 immunoreactivity, TUNEL sections were destained with 1% hydrochloric acid in 70% ethanol, followed by an antigen retrieval method with citrate buffer treatment in a microwave oven. The sections were then immunostained as described above.

## Results and Discussion

**Figure 1:**
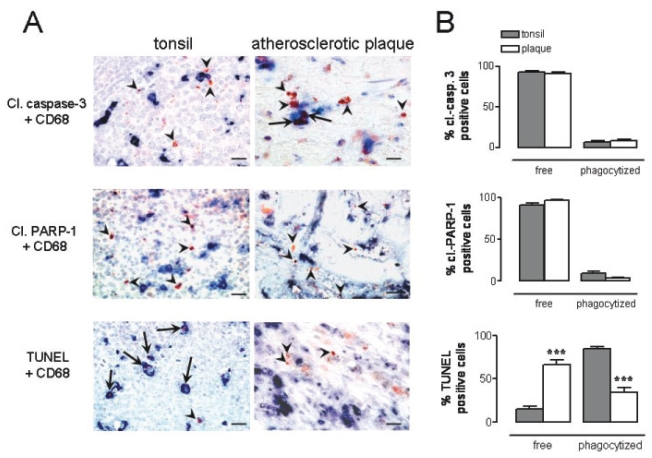
Phagocytosis of apoptotic cells (AC) by macrophages in human tonsils and atherosclerotic plaques. (**A**) Tissues were immunostained for macrophages (CD68, blue). Apoptotic cells (red-brown) were visualized either via immunohistochemical detection of cleaved caspase-3 or cleaved PARP-1, or via TUNEL. Arrowheads show free AC that have not been phagocytized by macrophages, whereas arrows indicate uptake of AC by macrophages. Both in tonsils and atherosclerotic plaques the majority of cleaved caspase-3 and cleaved PARP-1 positive AC were located outside but still in close proximity of macrophages. TUNEL positive AC in tonsils were found almost exclusively inside macrophages. In contrast, the majority of TUNEL-positive AC in atherosclerotic plaques was not phagocytized. (**B**) Quantification of free and phagocytized apoptotic cells in tonsils (grey bars, n = 8) and atherosclerotic plaques (open bars, n = 10). ***p < 0.001 vs tonsils (Mann-Whitney U test). Bar = 20 μm.

**Figure 2:**
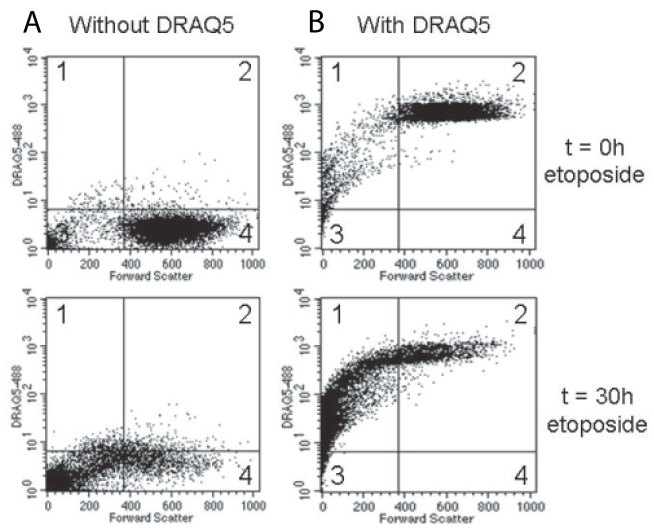
Flow cytometric analysis of etoposide-induced apoptosis in U937 monocytes. Cells were treated with 10 μM etoposide for 0–30 hours and were then analysed via flow cytometry in the absence (**A**) or presence (**B**) of 10 μM DRAQ5. Forward scatter in the X-axis is a measure for the size of cells and allowed us to distinguish viable cells from apoptotic bodies (high versus low forward scatter, respectively). Each dot plot consists of 4 quadrants representing following populations: DRAQ5 positive apoptotic cells (quadrant 1), DRAQ5 positive viable cells (quadrant 2), DRAQ5 negative apoptotic cells (quadrant 3) and DRAQ5 negative viable cells (quadrant 4). Incubations in the absence of DRAQ5 served as negative control for red fluorescence. After 30 hours of etoposide treatment, the majority of apoptotic cells (>95%) stained positive for DRAQ5 (quadrant 1).

Human tonsils and atherosclerotic plaques were immunostained for CD68, a cell surface marker which is present on macrophages, in combination with (immuno-) histochemical detection of an in situ marker for apoptosis such as cleaved caspase-3, cleaved PARP-1 or TUNEL. The number of TUNEL-positive AC in whole mount sections of carotid plaque specimens amounted to 85 ± 10. In addition, numerous cleaved PARP-1 and cleaved caspase-3 positive cells were detected (53 ± 3 and 48 ± 8 per mm^2^, respectively). In tonsils, we counted per germinal center 17 ± 2 TUNEL-positive AC, 71 ± 13 cleaved PARP-1 positive AC and 79 ± 8 cleaved caspase-3 positive AC. Both in tonsils and atherosclerotic plaques the majority of cleaved caspase-3 and cleaved PARP-1 positive AC were located outside but still in close proximity of macrophages (Figure 1). TUNEL positive AC in tonsils were found almost exclusively inside macrophages (Figure 1). However, the majority of TUNEL-positive AC in atherosclerotic plaques was not phagocytized, confirming recent data that phagocytosis of AC by plaque macrophages is severely impaired. In vitro experiments with U937 monocytes undergoing apoptosis after etoposide treatment showed that the majority of apoptotic cells (>95%) contain nuclear DNA and thus can be detected by TUNEL (Figure 2). We may therefore conclude that TUNEL, but not cleaved caspase-3 and PARP-1, in combination with macrophage immunostaining is a suitable method for evaluation of phagocytosis of AC by macrophages.

**Figure 3:**
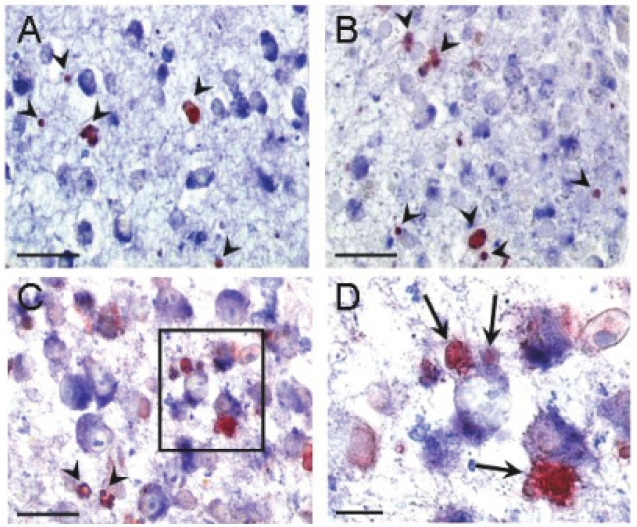
In vitro study of phagocytosis of apoptotic U937 cells by J774 macrophages. Macrophages were incubated with U937 cells (ratio 10:1) in the presence of 50 μM etoposide for 5 hours to allow induction of apoptosis of U937 cells followed by phagocytosis by J774 macrophages. Immunostaining for macrophages (Mac-3, blue) combined with cleaved PARP-1 (**A**, red), cleaved caspase-3 (**B**, red) or TUNEL (**C**, red) were performed. Arrowheads indicate free apoptotic cells. Bar = 20 μm. **D**, Magnification of the boxed area in panel **C** shows clear attachment and uptake of AC by macrophages (arrows). Bar = 10 μm.

To reinforce our in situ findings, co-cultures of J774A.1 macrophages and U937 cells (ratio 1:3) were treated with etoposide to selectively induce U937 apoptosis. After 5 hours, cells were embedded in paraffin, sectioned and immunostained in the same way as tissue. Cleaved PARP-1 and caspase-3 positive AC were not phagocytized by macrophages as shown for tissue (Figure 3A,B). However, in contrast to apoptotic cells in tonsils or atherosclerotic plaques, most TUNEL positive U937 cells were not phagocytized by macrophages (Figure 3C, arrows). Although this would be indicative of poor phagocytosis, preliminary experiments showed that phagocytosis should occur within the timeframe studied. Moreover, etoposide did not affect the phagocytosis capacity of the macrophage (data not shown). It is likely that the macrophages were overwhelmed by the massive induction of apoptosis in U937 cells. Indeed, by increasing the ratio of macrophages versus AC from 1:3 to 10:1, TUNEL-positive U937 cells, but not cleaved PARP-1 or caspase-3-positive cells, could be readily detected inside macrophages (Figure 3D). This limitation should be taken into account when studying phagocytosis of AC in vitro.

**Figure 4:**
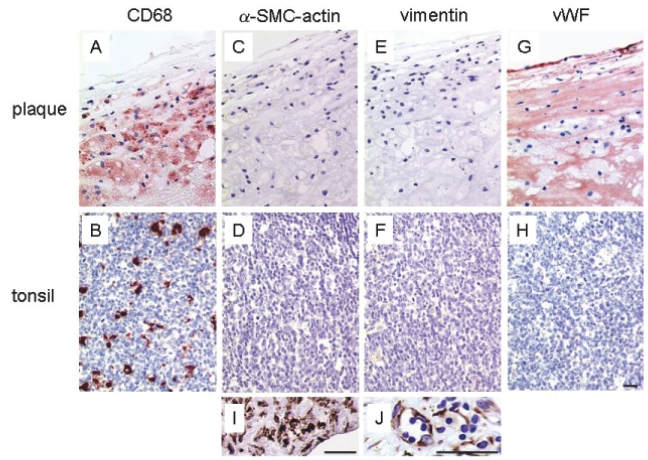
Immunohistochemical analysis of human carotid plaque (upper panels) and tonsil (lower panels) using cell type specific markers. The anti-CD68 antibody (PG-M1) strongly stained macrophages (red) in the plaque (**A**) and in the tonsil (**B**). The same regions did not show reactivity with the smooth muscle cell marker α-SMC-actin (**C**–**D**) and the fibroblast marker vimentin (**E**–**F**). The anti-von Willibrand Factor antibody clearly stained the endothelial cells (red) in the plaque (**G**) whereas germinal centers in tonsils (**H**) were negative. Panels **I** and **J** respectively showed an area with positive α-SMC-actin staining in the plaque and positive vimentin staining in the tonsil that did not colocalize with CD68 positive cells. Bar = 30 μm.

It is important to note that the TUNEL technique is easily prone to false positive staining, most notably after enhanced transcription (Kockx et al. 1998) or during induction of necrotic cell death (de Torres et al. 1997; Grasl-Kraupp et al. 1995; van Lookeren and Gill, 1996). If results are difficult to interpret, tissue analysis by electron microscopy is recommended to examine morphological features of apoptotic cells. Also of note, anti-CD68 antibodies are not macrophage-specific but may crossreact with fibroblasts and activated endothelial cells (Beranek, 2005; Kunisch et al. 2004). Moreover, CD68 is expressed in retinal epithelial cells, vascular smooth muscle cells, osteoblasts and fibroblast-like cells from the bone marrow. Since cross-reactivity depends on the epitope that is recognized by the antibody, stainings patterns may differ when using different anti-CD68 clones. Anti-CD68 clone PG-M1 used in this study exhibits the lowest crossreactivity with other cell types (0% of endothelial cells and less than 2% of synovial, gingival and skin fibroblasts) (Kunisch et al. 2004) and may therefore be the best choice to identify macrophages in different human tissues. To exclude nonspecific staining in human plaques and tonsils, we identified CD68 positive cells more thoroughly using other cell type specific markers (Figure 4). Anti-vimentin antibody, which is commonly used to identify fibroblasts, did not stain CD68 positive cells in carotid plaques or tonsil specimens. Similar results were obtained with antibodies directed against von Willebrand Factor and α-SMC-actin which are routinely used for the detection of endothelial cells and smooth muscle cells, respectively. It should be noted that during atherogenesis endothelial cells show enhanced vWF deposition (De Meyer et al. 1999), which may explain the additional staining of the subendothelial layer of the plaque (Figure 4G).

Apoptotic DNA degradation, as detected by TUNEL, may occur via two different mechanisms: within AC by caspase-activated DNase (CAD) and/or within the phagocyte by lysosomal DNase II after phagocytosis of AC (Nagata et al. 2003). Unlike DNase II, DNase I does not play a significant role in apoptotic DNA laddering as DNase I^−/−^ mice do not display altered chromatin breakdown during apoptosis (Napirei et al. 2004). DNase I is mainly found in body fluids such as serum and urine. It degrades chromatin in necrotic cells and has a main function in breakdown of dietary DNA within the alimentary tract (Napirei et al. 2004). Colocalization studies indicate that only a small fraction of the TUNEL-labeled nuclei in tonsils were cleaved caspase-3 positive (data not shown). This finding suggests rapid breakdown of cleaved caspase-3 (and probably also cleaved PARP-1) by phagosomal pH or lysosomal enzymes, once the AC is engulfed by the macrophage. On the other hand, TUNEL reactivity of AC can be a consequence of engulfment by the macrophage and degradation of the genomic DNA by DNase II. Both theories support the general assumption that TUNEL is the best method to detect phagocytosis of AC in situ. Another explanation for the lack of colocalization between TUNEL and cleaved caspase-3/PARP-1 immunostaining is the time lag between caspase-3 activation and CAD-dependent DNA fragmentation. One may assume that macrophages are able to recognize AC in a very early stage of apoptosis so that AC are engulfed before DNA fragmentation occurs. In line with this theory, Denecker et al. (2000) demonstrated that cell membrane properties that serve as eat-me signals (e.g. exposure of PS) change immediately after initiation of apoptosis, thus before cytochrome c release and activation of caspases can be detected. However, others showed that PS exposure on the apoptotic cell is blocked by the pan-caspase inhibitor z-VAD-fmk and therefore lies downstream from caspase activation. Moreover, these findings are cell culture observations. It remains unclear how efficient AC are recognized in tissue. As yet, we have no evidence for early recognition of AC in plaques or tonsils.

In conclusion, unremoved TUNEL-positive AC represent a pathological condition and are indicative of poor phagocytosis by macrophages. Other markers for apoptosis, such as cleavage of caspase-3 or PARP-1, should not be used to assess phagocytosis efficiency, since activation of the caspase cascade and cleavage of their substrates can occur in AC when they have not yet been phagocytized by macrophages.

## References

[b1-bmi-2006-193] Baumann I Kolowos W Voll RE 2002 Impaired uptake of apoptotic cells into tingible body macrophages in germinal centers of patients with systemic lupus erythematosus Arthritis Rheum 46 191 201 11817590 10.1002/1529-0131(200201)46:1<191::AID-ART10027>3.0.CO;2-K

[b2-bmi-2006-193] Beranek JT 2005 CD68 is not a macrophage-specific antigen Ann Rheum Dis 64 342 3 15647451 PMC1755344

[b3-bmi-2006-193] De Meyer GRY Hoylaerts MF Kockx MM 1999 Intimal deposition of functional von Willebrand factor in atherogenesis Arterioscler Thromb Vasc Biol 19 2524 34 10521383 10.1161/01.atv.19.10.2524

[b4-bmi-2006-193] de Torres C Munell F Ferrer I 1997 Identification of necrotic cell death by the TUNEL assay in the hypoxic-ischemic neonatal rat brain Neurosci Lett 230 1 4 9259449 10.1016/s0304-3940(97)00445-x

[b5-bmi-2006-193] Denecker G Dooms H Van Loo G 2000 Phosphatidyl serine exposure during apoptosis precedes release of cytochrome c and decrease in mitochondrial transmembrane potential FEBS Lett 465 47 52 10620704 10.1016/s0014-5793(99)01702-0

[b6-bmi-2006-193] Fadok VA Bratton DL Henson PM 2001 Phagocyte receptors for apoptotic cells: recognition, uptake, and consequences J Clin Invest 108 957 962 11581295 10.1172/JCI14122 PMC200959

[b7-bmi-2006-193] Grasl-Kraupp B Ruttkay-Nedecky B Koudelka H 1995 In situ detection of fragmented DNA (TUNEL assay) fails to discriminate among apoptosis, necrosis, and autolytic cell death: a cautionary note Hepatology 21 1465 8 7737654 10.1002/hep.1840210534

[b8-bmi-2006-193] Hodge S Hodge G Scicchitano R 2003 Alveolar macrophages from subjects with chronic obstructive pulmonary disease are deficient in their ability to phagocytose apoptotic airway epithelial cells Immunol Cell Biol 81 289 96 12848850 10.1046/j.1440-1711.2003.t01-1-01170.x

[b9-bmi-2006-193] Hogquist KA Nett MA Unanue ER 1991 Interleukin 1 is processed and released during apoptosis Proc Natl Acad Sci USA 88 8485 9 1924307 10.1073/pnas.88.19.8485 PMC52533

[b10-bmi-2006-193] Kockx MM Muhring J Knaapen MW 1998 RNA synthesis and splicing interferes with DNA in situ end labeling techniques used to detect apoptosis Am J Pathol 152 885 8 9546348 PMC1858249

[b11-bmi-2006-193] Kunisch E Fuhrmann R Roth A 2004 Macrophage specificity of three anti-CD68 monoclonal antibodies (KP1, EBM11, and PGM1) widely used for immunohistochemistry and flow cytometry Ann Rheum Dis 63 774 84 15194571 10.1136/ard.2003.013029 PMC1755048

[b12-bmi-2006-193] Lauber K Bohn E Krober SM 2003 Apoptotic cells induce migration of phagocytes via caspase-3-mediated release of a lipid attraction signal Cell 113 717 30 12809603 10.1016/s0092-8674(03)00422-7

[b13-bmi-2006-193] Nagata S Nagase H Kawane H 2003 Degradation of chromosomal DNA occurs during apoptosis Cell Death Differ 10 108 16 12655299 10.1038/sj.cdd.4401161

[b14-bmi-2006-193] Nakamura M Yagi H Kayaba S 1996 Death of germinal center B cells without DNA fragmentation Eur J Immunol 26 1211 6 8647194 10.1002/eji.1830260604

[b15-bmi-2006-193] Napirei M Wulf S Mannherz HG 2004 Chromatin breakdown during necrosis by serum Dnase1 and the plasminogen system Arthritis Rheum 50 1873 83 15188364 10.1002/art.20267

[b16-bmi-2006-193] Ren Y Tang J Mok MY 2003 Increased apoptotic neutrophils and macrophages and impaired macrophage phagocytic clearance of apoptotic neutrophils in systemic lupus erythematosus Arthritis Rheum 48 2888 97 14558095 10.1002/art.11237

[b17-bmi-2006-193] Schrijvers DM De Meyer GRY Kockx MM 2005 Phagocytosis of apoptotic cells by macrophages is impaired in atherosclerosis Arterioscler Thromb Vasc Biol 25 1256 61 15831805 10.1161/01.ATV.0000166517.18801.a7

[b18-bmi-2006-193] Schrijvers DM Martinet W De Meyer GRY 2004 Flow cytometric evaluation of a model for phagocytosis of cells undergoing apoptosis J Immunol Methods 287 101 8 15099759 10.1016/j.jim.2004.01.013

[b19-bmi-2006-193] van Lookeren CM Gill R 1996 Ultrastructural morphological changes are not characteristic of apoptotic cell death following focal cerebral ischaemia in the rat Neurosci Lett 213 111 4 8858621 10.1016/0304-3940(96)12839-1

[b20-bmi-2006-193] Vandivier RW Fadok VA Hoffmann PR 2002 Elastase-mediated phosphatidylserine receptor cleavage impairs apoptotic cell clearance in cystic fibrosis and bronchiectasis J Clin Invest 109 661 70 11877474 10.1172/JCI13572 PMC150889

[b21-bmi-2006-193] Woo M Hakem R Soengas MS 1998 Essential contribution of caspase 3/CPP32 to apoptosis and its associated nuclear changes Genes Dev 12 806 19 9512515 10.1101/gad.12.6.806 PMC316633

[b22-bmi-2006-193] Wyllie AH 1992 Apoptosis and the regulation of cell numbers in normal and neoplastic tissues: an overview Cancer Metastasis Rev 11 95 103 1394797 10.1007/BF00048057

